# Validation of Synthetic CRISPR Reagents as a Tool for Arrayed Functional Genomic Screening

**DOI:** 10.1371/journal.pone.0168968

**Published:** 2016-12-28

**Authors:** Jenille Tan, Scott E. Martin

**Affiliations:** Department of Discovery Oncology, Genentech, South San Francisco, CA, United States of America; Osaka University, JAPAN

## Abstract

To date, lentiviral-based CRISPR-Cas9 screens have largely been conducted in pooled format. However, numerous assays are not amenable to pooled approaches, and lentiviral screening in arrayed format presents many challenges. We sought to examine synthetic CRISPR reagents in the context of arrayed screening. Experiments were performed using aberrant DNA replication as an assay. Using synthetic CRISPR RNAs targeting the known control gene *GMNN* in HCT-116 cells stably expressing Cas9, we observed statistically significant phenotype among the majority of transfected cells within 72 hours. Additional studies revealed near complete loss of GMNN protein and editing of *GMNN* DNA. We next conducted a screen of synthetic CRISPR RNAs directed against 640 ubiquitin-related genes. Screening identified known and novel DNA replication regulators that were also supported by siRNA gene knockdown. Notably, CRISPR screening identified more statistically significant hits than corresponding siRNA screens run in parallel. These results highlight the possibility of using synthetic CRISPR reagents as an arrayed screening tool.

## Introduction

The ability to harness RNAi for functional genomics screening has improved our understanding of biology. However, the full potential of this technology is undermined by a high rate of false positives. It has been well established that false positives primarily arise from seed-based off-target effects[1]. Many computational and experimental strategies have been devised to overcome this problem[2–4]. However, none offer a comprehensive solution to off-target effects, and the ultimate outcome of most RNAi screens is an extensive list of candidate hits with many false positives.

The CRISPR-Cas9 system enables gene editing and target knockout, rather than post-transcriptional reduction of target mRNA, as with RNAi reagents. Initial efforts with the CRISPR-Cas9 system have suggested that it is less prone to off-target effects than RNAi[5, 6]. Like RNAi, CRISPR can be used for genome-wide screening (reviewed in [7]). To date, CRISPR-Cas9 screens have largely been conducted in pooled format. Pooled vector-based screening is a powerful approach that involves transducing Cas9 expressing cells with lentiviral constructs harboring single guide RNA (sgRNA), which is a chimera of the CRISPR-Cas9 system CRISPR RNA (crRNA) and trans-activating crRNA (tracrRNA)[8]. Cells are transduced such that each cell receives only one sgRNA. Once inside the cell, sgRNA can guide Cas9 to target DNA for editing. Editing leads to indel formation and the potential for functional knockout of targeted genes. A transduced pool of cells can then be subjected to selective pressure and sgRNAs that are enriched or depleted can be identified through next-generation sequencing.

Pooled screens are well suited for growth competition studies. For example, a pooled approach can be used to identify essential genes, or those that are synthetic lethal in the context of specific genotypes[6, 9–12]. Similarly, a pooled approach can be used to find genes that either enhance or mitigate the effect of a selective pressure or stimuli (e.g., rescue from virus-induced cell death[13–16]). One can also use strategies that employ cell sorting to identify a desired phenotype from pooled format (e.g., gain or loss of a reporter protein)[17–19]. However, there are many assays that are not amenable to pooled approaches, including a variety of high-content assays. For example, it would be difficult to use pooled approaches to study protein translocation from one compartment to another in a cell, or to detect low-level analytes that require more sensitive means of detection. siRNA screening has historically been used to investigate questions that can only be interrogated in arrayed format (one reagent per microplate well). Given the comparative advantages of CRISPR over RNAi technologies, we sought to examine CRISPR in the context of arrayed screening.

**Fig 1:**
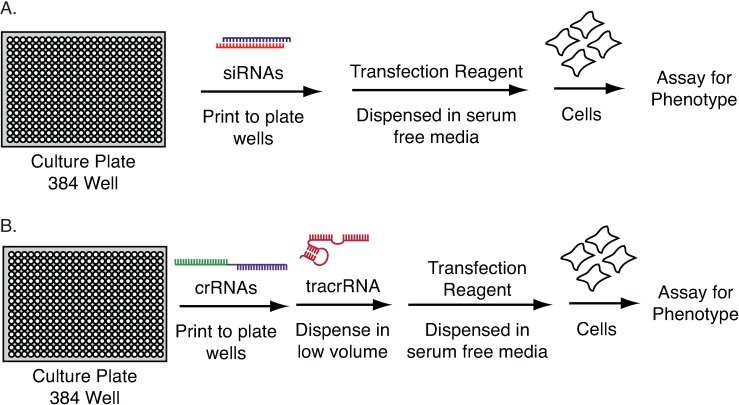
Workflows for arrayed screening in microplate format. (A) Reverse transfection of cells with siRNA. (B) Potential workflow for the reverse transfection of synthetic CRISPR reagents. crRNAs are first pre-spotted to plates and tracrRNA is added in serum free media. The complex is then incubated with lipid-based transfection reagents prior to the addition of cells.

Lentiviral-based screening is not easily applied in microplate format, although it has been reported[20–22]. Large-scale manipulations of virus across hundreds of plates present a variety of challenges, including safety and variable well-to-well titer. Moreover, the time needed for sgRNA expression, subsequent editing, and gene product turnover may be too long for microplate assays. Synthetic siRNAs overcome these issues in the context of RNAi screening due to the rapidity of mRNA targeting and degradation. They can be pre-spotted to microplates and subsequently reverse transfected into cells (Fig 1A). This approach is easily scaled for genome-wide screens.

In principle, complexes of synthetic crRNA and tracrRNA could be used in much the same way as siRNA (Fig 1B). However, there are many questions regarding this approach. A major concern is editing efficiency, as low rates of loss-of-function editing could mean that a large fraction of cells would remain unchanged. This would reduce signal to noise in any assay, and likely be most pronounced in growth assays where unedited cells could outgrow those that have been edited. Even with highly efficient editing, it may take too long to practically conduct screens in microplate format. In light of these questions, we set out to evaluate synthetic CRISPR screening using aberrant DNA replication as a readout. Effects on DNA replication can be easily measured through changes in DNA stain intensity and nuclear area by automated microscopy. Cell-by-cell analysis permits the identification of events exhibited by only a fraction of cell in a population, so that effects can be observed even in cases of low editing efficiency.

Using synthetic crRNAs targeting *GMNN*, a gene known to be critical for DNA replication[23], we observed aberrant nuclei in the majority of transfected cells. Surprisingly, effects were observed in 72 hours post-transfection. Additional experiments revealed significant loss of GMNN protein and editing of *GMNN* DNA. We next conducted a screen of synthetic CRISPR RNAs directed against 640 ubiquitin-related genes, including E3 ligases and deubiquitinating enzymes. Synthetic CRISPR screening identified known and novel genes associated with DNA replication that were also supported by siRNA reagents. Notably, CRISPR screening identified a greater number of statistically significant hits than corresponding siRNA screens, as the correlation between different synthetic CRISPRs targeting the same gene was better than that observed with siRNAs. These results validate the use of synthetic CRISPR as a tool for arrayed screening in amenable assay systems.

## Results

**Fig 2:**
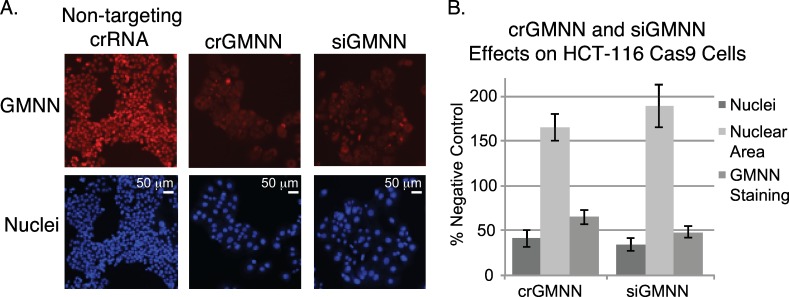
Cellular effects of modulating *GMNN* with synthetic CRISPR reagents or siRNA. (A) Representative images illustrating alterations in nuclear size (Hoechst staining; lower panels) and GMNN protein levels by immunofluorescence (upper panels). (B) Quantitation of changes in nuclear count, nuclear area, and GMNN protein levels (n = 8 per condition; median effect and standard deviation are represented).

We first obtained HCT-116 cells stably expressing Cas9 protein to evaluate synthetic CRISPR, as the knockdown of a several control genes, including *GMNN*, results in enlarged nuclei with increased DNA content in HCT-116 cells[23, 24]. HCT-116 Cas9 cells were then reverse transfected with crRNA:tracrRNA complexes targeting *GMNN* and monitored for changes in nuclear size. Many enlarged nuclei were observed within 72 hours (Fig 2). The effects were comparable to those observed with siRNA in terms of the average increase in nuclear area (Fig 2). Immunofluorescence also indicated a corresponding reduction of GMNN protein levels (Fig 2). No effect was observed in HCT-116 cells transfected with crRNA:tracrRNA lacking Cas9 (S1 Fig).

**Fig 3:**
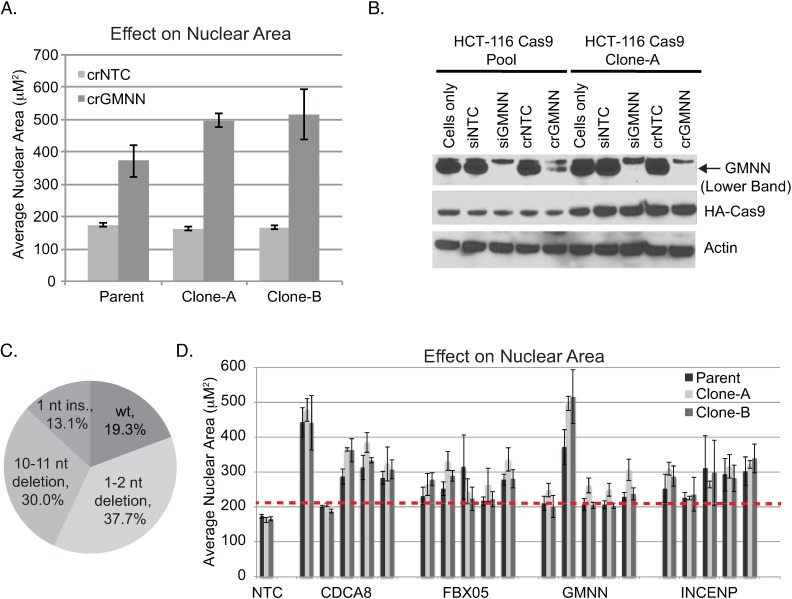
Selection and characterization of HCT-116 Cas9 clones. (A) Clones transfected with synthetic crRNA targeting *GMNN* exhibit increased nuclear area as compared to the parental HCT-116 Cas9 population. All exhibited a significant increase for crGMNN versus non-targeting control (p < 0.05) (B) Western blot indicates a greater decrease in GMNN protein for clones transfected with crGMNN versus parental HCT-116 Cas9 cells. Clones also exhibit greater levels of Cas9. (C) NGS sequencing of the *GMNN* target region in transfected cells (Clone-A) indicates >80% editing of *GMNN* DNA. (D) Effects of crRNAs targeting control genes known to affect nuclear area in HCT-116 Cas9 polyclonal and clonal populations. Bars represent the average and standard deviation of four replicates. The dashed line indicates five standard deviations above non-targeting control. Data for additional genes can be found in S2 Fig.

Although GMNN crRNA yielded clear phenotypes within 72 hours post-transfection, there was a fraction of cells whose nuclear size appeared to be unaltered. We reasoned that these cells may be less prone to editing. Accordingly, we selected single cell clones and evaluated them with crRNA targeting *GMNN*. A number of clones exhibited more penetrant phenotypes than parental polyclonal HCT-116 Cas9 cells, as judged by an overall increase in nuclear area and the fraction of significantly enlarged cells (Fig 3A and S2 Fig). These clones also exhibited a greater reduction in GMNN protein levels and higher levels of Cas9 protein (Fig 3B). Sequencing of cells treated with crRNA:tracrRNA targeting *GMNN* also demonstrated editing of *GMNN* DNA in > 80% of templates (Fig 3C).

We evaluated additional crRNAs targeting several genes known to yield enlarged nuclei and increased DNA stain intensity upon knockdown in HCT-116 cells[24]. Five crRNAs were tested per gene. Multiple crRNAs per gene yielded a significant increase in average nuclear area as compared to non-targeting control (> 5 standard deviations above non-targeting control, Fig 3D and S3 Fig). However, crRNAs for a number of genes failed to yield a significant effect, and the magnitude of response for active crRNAs targeting the same gene was considerably different in some cases. For example, *GMNN*, *CDCA8*, and *AURKB* all had one corresponding crRNA that appreciably outperformed the others.

Many proteins involved in DNA replication are tightly controlled by the ubiquitin/proteasome system, and so we next conducted a screen for ubiquitin/proteasome genes that affect DNA replication. The screen employed a library targeting 640 ubiquitin-related genes (E1 activating enzymes, E2 conjugating enzymes, and E3 ligases, as well as deubiquitinating enzymes) with 4 crRNAs per gene. Screening was conducted using an HCT-116 Cas9 clone that displayed a relatively strong response to crRNA targeting *GMNN* (Clone-A). The cells were reverse transfected in 384 well plate. Reverse transfection is highly amenable to automation and easily scaled for genome-wide screens (Fig 1B). The cells were fixed 96 hours post-transfection, and stained (Hoechst) for imaging and analysis. siRNA screening was conducted in parallel with a library targeting the same 640 genes with four siRNAs per gene, one siRNA per well (GE Dharmacon On-Target Plus). crRNA and siRNA screens were assayed in biological duplicate and performed using the same clonal population of HCT-116 Cas9 cells.

**Fig 4:**
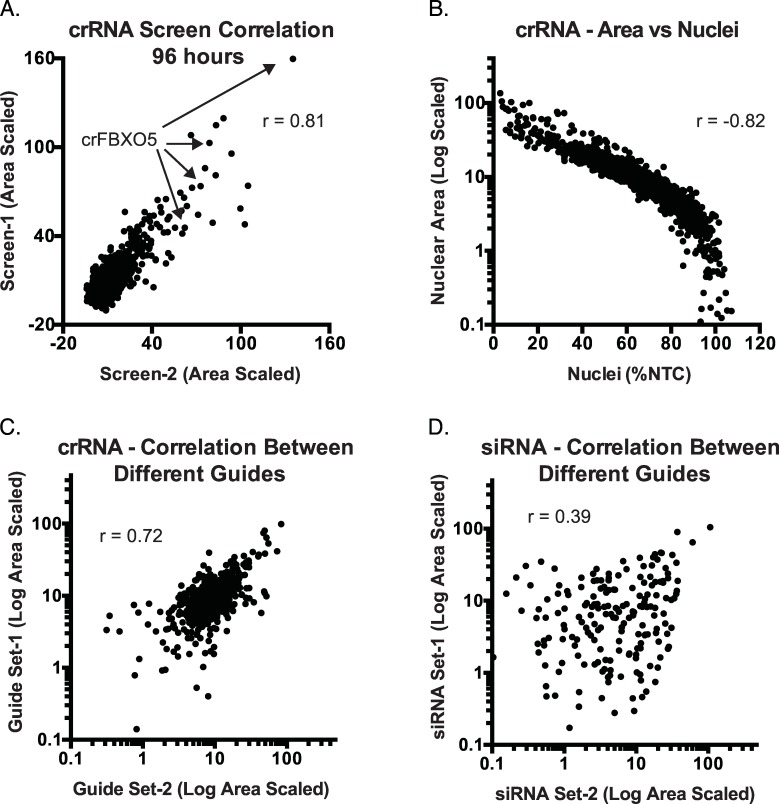
Various relationships among crRNA and siRNA screen data. (A) Biological replicates of a synthetic CRISPR screening experiment. Each dot indicates the value derived from a single crRNA assayed in separate experiments. (B) Increased nuclear area exhibits a strong negative correlation with nuclear count. Points correspond to values from individual wells. (C-D). Comparison of the effect of different guides (C) or siRNA (D) targeting the same gene on measured nuclear area. “Sets” are the average activity of randomly selected pairs of guides or siRNAs targeting the same gene. There are two pairs per gene given that each gene has 4 corresponding crRNA or siRNA reagents. The correlation between any randomly selected single crRNA targeting the same gene was also greater than that for siRNA (r = 0.45 versus r = 0.18; S4 Fig).

Screening was conducted with non-targeting crRNAs and with crRNAs targeting genes known to influence nuclear area (*GMNN*) and nuclear count (*PLK1)* as positive controls. Control crRNAs exhibited assay z’-factors of ≥ 0.5 for both *GMNN* and *PLK1* crRNAs. Replicate screens also correlated well in terms of average nuclear area (r = 0.81, Fig 4A). Nuclear area also exhibited a strong negative correlation with nuclear count (r = -0.82, Fig 4B) and DNA stain intensity (r = 0.91), suggesting that these are largely surrogate readouts. Importantly, different crRNAs targeting the same gene were active in many cases (Fig 4C). For example, multiple crRNAs targeting the integral cell cycle regulator *FBXO5[25]* increased nuclear area to a level comparable to that of *GMNN* crRNA (Fig 4A). *FBXO5* is an F-box protein subunit of the SCF complex, and multiple crRNAs targeting other subunits of the SCF complex, *RBX1* and *CUL1*, were also active. siRNA assay z’-factors for siGMNN an siPLK1 controls were also high in siRNA screens (> 0.5), and identified genes with multiple active siRNAs. However, different siRNAs targeting the same gene had a more variable impact on nuclear area than that observed between different crRNAs (Fig 4D).

**Fig 5:**
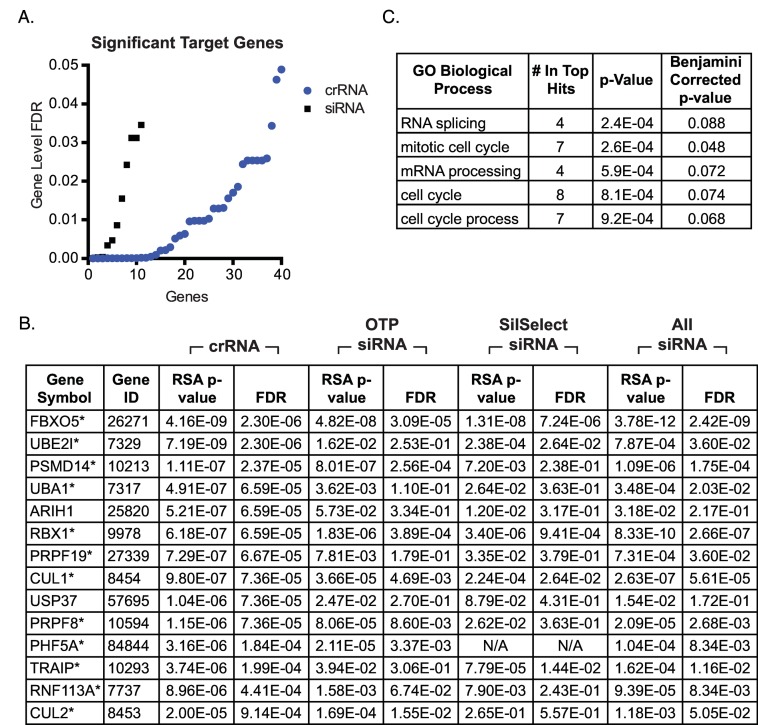
Evaluation of screen results from synthetic CRISPR and siRNA screens of ubiquitin system genes. (A) RSA reveals many more significant hits (FDR < 0.05) for CRISPR relative to siRNA screening. (B) Genes identified through CRISPR screening with a RSA corrected p-values < 0.001 and corresponding values from siRNA screening. “OTP siRNA” refers to results from screening of the Dharmacon On-Target Plus siRNA library. “SilSelect siRNA” refers to results from screening the Ambion Silencer Select siRNA library. “All siRNA” refers to results obtained after combing data from both siRNA screens. *Refers to crRNA screen hits that appear to be supported by siRNA data. (C) Genes with RSA corrected p-value < 0.001 in the CRISPR screen are associated with progression through the cell cycle.

Redundant siRNA analysis (RSA) was used to identify genes affecting nuclear area. RSA integrates the performance of all reagents targeting a given gene to impute the significance of its influence on the screening assay[26]. Overall, crRNA screening identified more significant genes involved in DNA replication than were observed in the On-Target Plus siRNA screen (Fig 5A and 5B; S2 and S3 Tables). At an FDR of 0.01, crRNA screening identified 24 genes (S2 Table), which were associated with DNA replication and cell cycle progression (Fig 5C). At the same FDR, siRNA screening identified only 6 genes, all of which were among the top candidates identified in the crRNA screen (S3 Table). Moreover, all but two (ARIH1 and USP37) of the top crRNA screen candidates (FDR = 0.001, 14 genes) were supported by siRNA data from the On-Target plus screen or data obtained from additional screen with an independent siRNA library (Fig 5B). The image-based phenotypes for well-scoring genes known to be associated with aberrant DNA replication, including *FBXO5*, *RBX1*, *CUL1*, and *TRAIP*[27, 28], were consistent with nuclear area measurements (S5 Fig).

## Discussion

The use of expression systems to introduce sgRNA has largely restricted CRISPR screens to pooled vector-based approaches. Synthetic CRISPR reagents provide a means to potentially overcome many of the issues associated with lentivirus-based screening in arrayed format. Our results suggest that large-scale arrayed screening with synthetic CRISPR reagents is feasible, and can be applied using a workflow analogous to that of high-throughput screening with siRNAs. Since gene targeting effects are observed in a matter of days, the results of synthetic crRNA screens may be more comparable to those obtained by siRNA screening than those obtained with long-term pooled assays. Similarly, phenotypes can be observed with synthetic crRNAs targeting essential genes that would be lost in long-term pooled CRISPR screens. A very recent study also supports our conclusions by demonstrating the utility of complexing synthetic crRNAs with recombinant Cas9 protein, and introducing the resulting ribonucleoproteins into primary T cells via electroporation[29]. This method was able to achieve functional knockout of control genes in roughly two thirds of transfected cell populations, and was subsequently used for the screening of 45 genes in 96 well format.

It may be difficult to apply synthetic CRISPR approaches to identify essential genes in an extended assay, because a fraction of the cell population will remain unedited and/or will retain enough functional protein to mask the effect of the deletion. Selecting more responsive clones from a population of Cas9-expressing cells may help to improve penetrance and the chances of observing activity. For example, HCT-116 Cas9 clones expressing higher levels of Cas9 protein exhibit more penetrant response than parental cells in response to *GMNN* and *PLK1* crRNAs. We have also observed similar improvements using clonal populations of Cas9 cells in other assays (data not shown). However, selecting clonal populations comes with its own risks, as a single clone may be non-representative of the parental population. This could lead to the identification of biology that has no relevance beyond the tested clone. Accordingly, it would be advisable to test multiple clones in follow-up validation experiments. As an alternative to clones, one could consider using GFP as a surrogate marker for Cas9 expression and enrich for populations with high GFP expression by FACS. Regardless, Cas9-expressing cell lines must be evaluated for the extent of editing efficiency and assay performance (e.g., assay z’ factor) prior to any screen, as with any assay.

Much is still being learned about the limitations of CRISPR screening. Arrayed screening with synthetic crRNA will certainly present its own challenges. For example, effective sequence design is an issue for both pooled and arrayed screens. Notably, a number of significant gene hits had inactive crRNAs, as did control genes known to yield enlarged nuclei in HCT-116 cells. Moreover, active sequences targeting the same gene exhibit varying magnitudes of effect, although the agreement between crRNA reagents targeting the same gene is largely better than that observed with siRNAs. False positives will also remain an issue for CRISPR-based screening. Recent work has demonstrated a correlation between phenotype and copy number that presumably arises as a function of DNA damage rather than on-target toxicity[30]. It may also be difficult to apply this type of strategy with high ploidy cell lines that require more editing events per cell for complete knockout, or other cell lines that are simply refractory to this type of approach. Nonetheless, a variety of studies have demonstrated more robust data with CRISPR screening versus RNAi[5, 6], which is supported by our observations that known replication control genes achieve greater statistical significance in synthetic CRISPR versus siRNA screening in arrayed format. Even a modest improvement in the fidelity of screen data compared to RNAi would significantly improve our ability to identify true positives in well-designed assays.

## Materials and Methods

### Tissue Culture

HCT-116 cells were obtained from ATCC and maintained by an internal cell line repository, which ensures all cell lines undergo rigorous authentication and quality controls. The controls include:

### Short Tandem Repeat (STR) Profiling

STR profiles are determined for each line using the Promega PowerPlex 16 System. This is performed once and compared to external STR profiles of cell lines (when available) to determine cell line ancestry. **Loci analyzed:** Detection of sixteen loci (fifteen STR loci and Amelogenin for gender identification), including D3S1358, TH01, D21S11, D18S51, Penta E, D5S818, D13S317, D7S820, D16S539, CSF1PO, Penta D, AMEL, vWA, D8S1179 and TPOX.

### SNP Fingerprinting

SNP profiles are performed each time new stocks are expanded for cryopreservation. Cell line identity is verified by high-throughput SNP profiling using Fluidigm multiplexed assays. SNPs were selected based on minor allele frequency and presence on commercial genotyping platforms. SNP profiles are compared to SNP calls from available internal and external data (when available) to determine or confirm ancestry. In cases where data is unavailable or cell line ancestry is questionable, DNA or cell lines are re-purchased to perform profiling to confirm cell line ancestry. SNPs analyzed: rs11746396, rs16928965, rs2172614, rs10050093, rs10828176, rs16888998, rs16999576, rs1912640, rs2355988, rs3125842, rs10018359, rs10410468, rs10834627, rs11083145, rs11100847, rs11638893, rs12537, rs1956898, rs2069492, rs10740186, rs12486048, rs13032222, rs1635191, rs17174920, rs2590442, rs2714679, rs2928432, rs2999156, rs10461909, rs11180435, rs1784232, rs3783412, rs10885378, rs1726254, rs2391691, rs3739422, rs10108245, rs1425916, rs1325922, rs1709795, rs1934395, rs2280916, rs2563263, rs10755578, rs1529192, rs2927899, rs2848745, rs10977980

### Mycoplasma Testing

All stocks are tested for mycoplasma prior to and after cells are cryopreserved. Two methods are used to avoid false positive/negative results: Lonza Mycoalert and Stratagene Mycosensor.

Cell growth rates and morphology are also monitored for any batch-to-batch changes.

### Cas9 Cell Generation

Cas9 was cloned into pLenti6.3 (Invitrogen, vector map provided as S1 Map) and lentiviral particles were generated using standard methods. Briefly, 1E7 293T cells were seeded in a 10cm culture dish along with growth media (DMEM, 10% FBS, 2mM L-glutamine, 100μM non-essential amino acids) for ~24 hours to reach 90%-95% confluence. 15.5μg of DNA mix comprising Cas9 expression plasmid, delta8.9 (packaging plasmid) and VSVG (envelope plasmid) was prepared at a molar ratio of 1:2.3:0.2 and diluted in 0.5mL of Opti-MEM I. Lipofectamine 2000 (ThermoFisher Scientific) reagent (2μL/μg DNA) was diluted in 0.5mL of Opti-MEM I medium and incubated at room temperature for 5 minutes. Diluted Lipofectamine 2000 and DNA were combined and incubated at room temperature for 20 minutes before adding to cells. Cells were then incubated at 37°C for 6 hours. Media containing the transfection mix was aspirated and replaced with 8 mL of growth medium, and incubated at 37°C for ~ 40 hours. Supernatant was collected, clarified by filtering through a 0.45μm filter, and concentrated by incubation with Lenti-X Concentrator (Clontech) at 4°C for 1 hour followed by centrifuging at 3100rpm in an Allegra X-12R tabletop centrifuge at 4°C for 45 minutes. HCT-116 cells were infected with an MOI of greater than 1, and stable integration was selected with 5 μg/mL blasticidin. HCT-116.pLENTI6.3-Cas9 cells were maintained in RPMI 1640 supplemented with 10% fetal bovine serum (Sigma), 2 mM L-glutamine, and 4 μg/ml Blasticin.

### Clonal Isolation and Selection

Clones of HCT 116.pLENTI6.3-Cas9 cells were generated by limiting dilution. Cells were grown for 2 weeks and monitored for single colonies. Each single colony was expanded and transfected. Gene editing activity was monitored in each clone by assessment of changes in nuclear area following transfection with crRNA targeting *GMNN*.

### Control siRNA and crRNA Sequences

GMNN crRNA; GE Dharmacon Edit-R, Sequence: AAAUCUUGGAGGAGUCACCC

PLK1 crRNA; GE Dharmacon Edit-R, Sequence: GAUCUCGGACGCGGACACCA

crNTC; GE Dharmacon Edit-R, Sequence: GUAACGCGAACUACGCGGGU

GMNN siRNA; Ambion Silencer Select, Cat ID:s27305 Target: GAAUAGUUCUGUCCCAAGATT

PLK1 siRNA; Ambion Silencer Select Cat ID:s448 Target: CCAUUAACGAGCUGCUUAATT

Silencer Select negative control siRNA #2 was used as the negative control for siRNA experiments. Additional crRNA control sequences used in Fig 3D can be found in S1 Table.

### Transfections

Optimized reverse transfection conditions for HCT 116.pLENTI6.3-Cas9 cells were determined using a 384-well plate by varying transfection reagent amount, Dharmacon EDIT-R crRNA concentration, and Dharmacon tracrRNA amount. Optimal conditions were found to be 0.08 μL Lipofectamine RNAiMAX (ThermoFisher Scientific), 20nM crRNA:tracrRNA, and 1500 cells/well in a total volume of 40μL. For screening, 0.8 pmol of crRNA is spotted onto a 384-well plate in 2 μL of water and allowed to complex with an equimolar amount of tracrRNA in serum free RPMI for 10 minutes at ambient temperature. 0.08 μL RNAiMax in 10 μL RPMI was then added to the complex. After a 30 minute incubation, 1500 cells/well were plated in 20% serum RPMI media. Transfections were incubated and monitored for 72–96 hours.

Significance in Fig 3A was determined by a two-tailed t-test assuming unequal variance.

### Screening

CRISPR screens were conducted using an Edit-R library (GE Dharmacon) of crRNAs targeting 640 ubiquitin-related genes with 4 crRNAs per gene. The library was pre-spotted to 384 well plates and transfected as described above. The screen was allowed to proceed for 96 h prior to fixing and staining (described below). Screening was also conducted for 72 h (data not shown), but effects were more pronounced at 96 h. An entire column (16 wells) of non-targeting and GMNN crRNAs were used as negative and positive controls. Images were acquired on an ImageXpress Micro XLS (Molecular Devices) and analyzed using associated MetaXpress software. Data was mapped and normalized using Genedata Screener software. Nuclear count and intensity were normalized to non-targeting control. Nuclear area was normalized using a scale set between non-targeting (0) and crGMNN (100) controls.

siRNA screening was conducted using an On-Target Plus library (GE Dharmacon) of siRNAs targeting a near identical set of genes as the crRNA library with 4 siRNAs per gene, one siRNA per well (all genes in the Edit-R library were represented in the OTP library). The screen was conducted in the same manner as for the crRNA library except that 0.8 pmol of siRNA was pre-spotted to plates and 0.08 μL of RNAiMax was added in 20 μL of serum free RPMI prior to the addition of 1500 cells in 20% serum RPMI media. siRNA screening was conducted for 72 hours, as siRNAs generally elicited phenotypes faster than crRNAs. An additional siRNA screen was conducted using a Silencer Select library (Life Technologies) targeting 1077 genes with 3 siRNAs per gene. 87% of genes in the Edit-R library were represented in the Silencer Select library.

All screens were performed using the same clonal population of HCT-116 Cas9 cells in biological duplicate, and the average results were used for analysis. Only data for the subset of genes found in the synthetic CRISPR library were used for RSA analysis of siRNA data. When examining the data from combined siRNA screens (Dharmacon OTP plus or Ambion Silencer Select), duplicate sequences found in the Ambion Silencer Select library that were already represented in the Dharmacon OTP library were removed prior to RSA analysis. All CRISPR and siRNA screening data can be found in S2–S5 Tables. FDR corrections of RSA p-values were performed in R data analysis software using Benjamini-Hochberg[31].

Pathway analysis was performed using DAVID[32, 33]. Genes with an FDR corrected RSA p-value < 0.01 were analyzed using all genes in the synthetic CRISPR screen as background.

### Western Blotting

Cells were lysed using RIPA lysis buffer with Halt Protease and Phosphatase Inhibitor Cocktail (ThermoFisher Scientific). Protein concentration was determined using the Bradford protein assay, and 20 μg of samples were loaded along with Nupage LDS Sample Buffer 4x (ThermoFisher Scientific) onto NuPAGE™ Novex™ 4-12% Bis-Tris Protein Gels (ThermoFisher Scientific). The protein was transferred onto a 0.45μm nitrocellulose membrane using a Trans-Blot Turbo Transfer System (Bio-Rad). Immunoblotting was performed with the following antibodies: 1/1000 anti-Geminin (Abcam ab12147), 1/1000 anti-HA (Covance MMS-101P), 1/2000 anti-Beta Actin HRP (CST 5125S), 1/1000 anti-mouse HRP (MP Biomedicals), 1/1000 anti-rabbit HRP (Jackson ImmunoResearch).

### Immunofluorescence and Imaging

Cells were fixed using 8% paraformaldehyde in PBS for 30 minutes and then washed three times with PBS. Cells were then permeabilized using 0.5% Saponin in Dulbecco’s PBS (ThermoFisher Scientific) for 15 minutes then washed twice with PBS. Wells were blocked using Block Buffer (30% BSA, 10x DAKO, water) for 1 hour and then washed twice with PBS. Immunoflourescence was performed with the following: 1/3000 Hoechst 33342 (ThermoFisher Scientific), 1/1000 anti-Geminin (Abcam ab12147), 1/500 anti-HA (Covance MMS-101P), Goat anti-Mouse IgG, Alexa Flour 488 (ThermoFisher Scientific), Donkey anti-Rabbit IgG, Alexa Flour 647 (ThermoFisher Scientific). Assay plates were imaged using a Molecular Devices ImageXpress Micro.

### PCR and Sequencing

PCR reactions contained 25 μl JumpStart Taq DNA Polymerase (Sigma-Aldrich), 0.3 μM of forward and reverse primers (Forward 5’-GGTGACAGAGCGAGACTCTAA-3’, Reverse 5-GCTTCAACCTCCTAAGCTACTG-3’), and 3 μl of 10 ng DNA extracted from transfected cells. The following thermal cycling steps were then run on the samples: 95°C for 10 minutes, followed by 30 cycles of 95°C for 10 seconds, 62°C for 30 seconds, and 68°C for 1 minute, and a final extension at 72°C for 7 minutes. PCR products were run on 2% E-Gel General Purpose Agarose Gels (ThermoFisher Scientific). For sequencing, PCR products were purified using the QIAquick PCR Purification Kit (Qiagen) and submitted for in-house sequencing.

### Next Generation Sequencing

A shorter length (361bp) amplicon of the gene edited region was generated using PCR reactions from above using 0.3 μM of forward and reverse primers (Forward 5’- AGGAAACATCGGAATGACCAC-3’, Reverse 5- GCTTCAACCTCCTAAGCTACTG-3’) and purified using the QIAquick PCR Purification Kit. Samples were then quantified using a Qubit Flourometer (ThermoFisher Scientific) and submitted for Amplicon Sequencing on the Illumina MiSeq using a 200 cycles sequencing run. GSNAP[34] was used to align the Illumina reads to the human reference genome GRCh38. Next, each read aligning to the target site was scanned for mutations and indels. All mutation events within a 50 bp window at the target site and occurring with a frequency above 0.01 were reported.

## Supporting Information

S1 Fig crRNAs have no effect on HCT-116 cells lacking Cas9 in terms of nuclear count or nuclear area. Bars equal average (n = 8) and standard deviation. siRNAs targeting GMNN and PLK1 exhibit expected effects under the same transfection conditions.
(PDF)

S2 Fig The majority of HCT-116 Cas9 cells are affected by crGMNN. (A) Histogram showing a shift in nuclear area for the majority of cells transfected with crRNA GMNN as compared to non-targeting crRNA in polyclonal HCT-116 cells. (B) Clonal HCT-116 cells shows an even greater fraction of cells with ~7x increased nuclear area. (C) Magnified version of the data in (B).
(PDF)

S3 Fig Effects of crRNAs targeting control genes known to affect nuclear area in HCT-116 Cas9 polyclonal and clonal populations. Bars represent the average and standard deviation of four replicates. The dashed line indicates five standard deviations above non-targeting control.
(PDF)

S4 Fig Comparison of the effect of different guides (left) or siRNAs (right) targeting the same gene on measured nuclear area. (PDF)

S5 Fig Effects of crRNAs targeting genes known to affect aberrant DNA replication. (PDF)

S1 Map Map of vector used to generate HCT-116 cells with stable expression of Cas9. (PDF)

S1 Table crRNA sequences for control genes used in Fig 3B. crRNAs are listed in the order they appear in the figure.
(XLSX)

S2 Table Dharmacon Edit-R crRNA screen data. (XLSX)

S3 Table Dharmacon OTP siRNA screen data. (XLSX)

S4 Table Ambion Silencer Select siRNA screen data. (XLSX)

S5 Table RSA results for combined siRNA screen data. Only genes that intersect with the crRNA library were considered and no duplicate sequences already found in the OTP library were included from the Ambion data.
(XLSX)
