# Supplementary material for: Validation of Synthetic CRISPR Reagents as a Tool for Arrayed Functional Genomic Screening
Source: PLoS One. 2016 Dec 28;11(12):e0168968. doi: 10.1371/journal.pone.0168968 (PMC5193459; doi:10.1371/journal.pone.0168968)

Supplemental Figure 1. crRNAs have no effect on HCT-116 cells lacking Cas9 in terms of nuclear count or nuclear area. Bars equal average (n=8) and standard deviation. siRNAs targeting GMNN and PLK1 exhibit expected effects under the same transfection conditions.

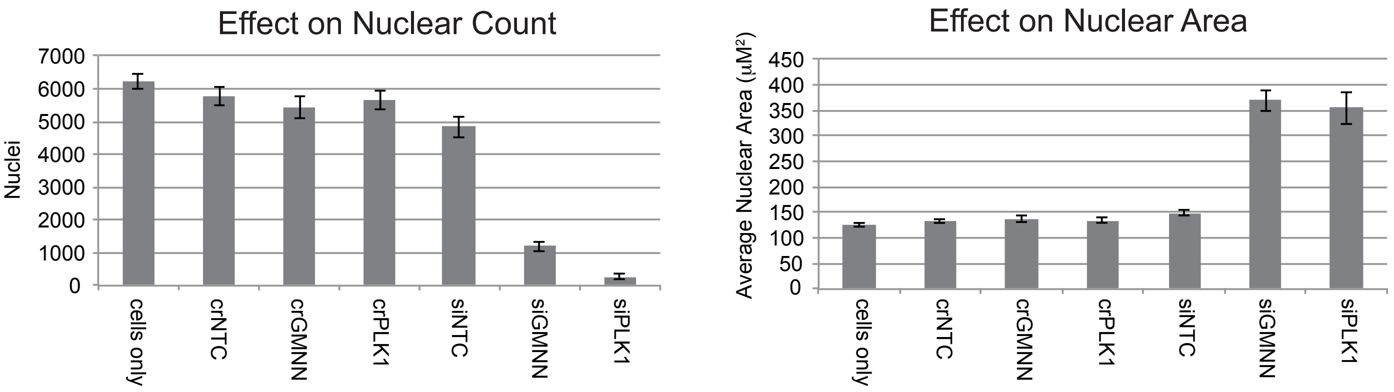

Supplement: S1 Fig — Bars equal average (n = 8) and standard deviation. siRNAs targeting GMNN and PLK1 exhibit expected effects under the same transfection conditions. (PDF) [file pone.0168968.s001.pdf]
