# Supplementary material for: Validation of Synthetic CRISPR Reagents as a Tool for Arrayed Functional Genomic Screening
Source: PLoS One. 2016 Dec 28;11(12):e0168968. doi: 10.1371/journal.pone.0168968 (PMC5193459; doi:10.1371/journal.pone.0168968)

Supplemental Figure 2. The majority of HCT-116 Cas9 cells are affected by crGMNN. A. Histogram showing a shift in nuclear area for the majority of cells transfected with crRNA GMNN as compared to non-targeting crRNA in polyclonal HCT-116 cells. B. Clonal HCT-116 cells shows an even greater fraction of cells with ~7x increased nuclear area. C. Magnified version of the data in B.

A.

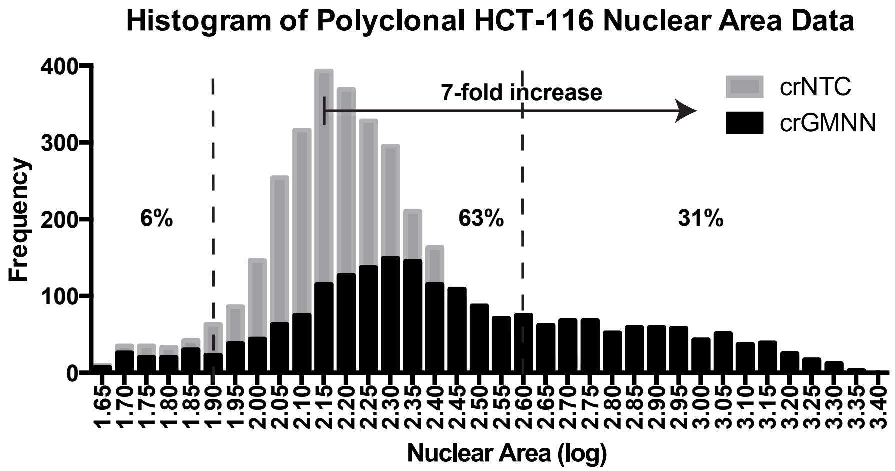

B.

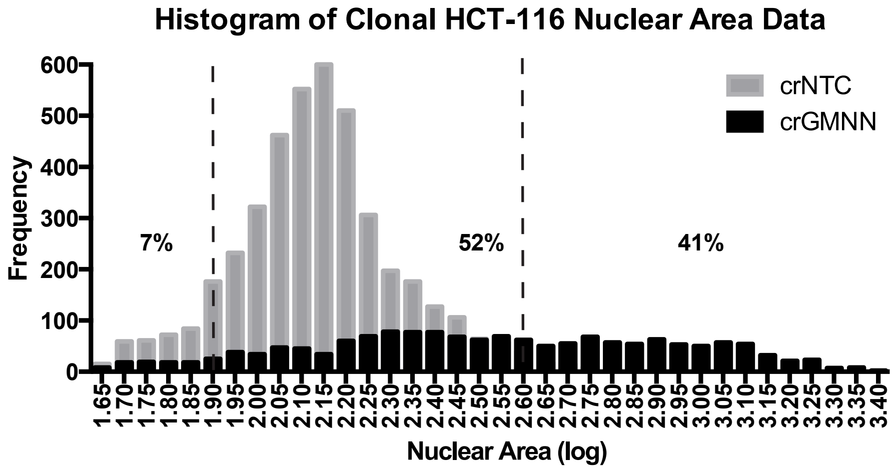

C.

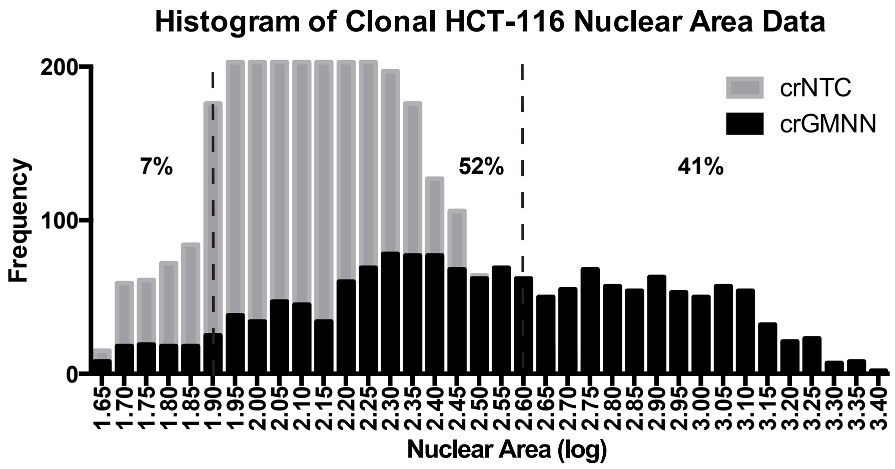

Supplement: S2 Fig — (A) Histogram showing a shift in nuclear area for the majority of cells transfected with crRNA GMNN as compared to non-targeting crRNA in polyclonal HCT-116 cells. (B) Clonal HCT-116 cells shows an even greater fraction of cells with ~7x increased nuclear area. (C) Magnified version of the data in (B). (PDF) [file pone.0168968.s002.pdf]
