# Supplementary material for: Validation of Synthetic CRISPR Reagents as a Tool for Arrayed Functional Genomic Screening
Source: PLoS One. 2016 Dec 28;11(12):e0168968. doi: 10.1371/journal.pone.0168968 (PMC5193459; doi:10.1371/journal.pone.0168968)

Supplemental Figure 3. Effects of crRNAs targeting control genes known to affect nuclear area in HCT-116 Cas9 polyclonal and clonal populations. Bars represent the average and standard deviation of four replicates. The dashed line indicates five standard deviations above non-targeting control.

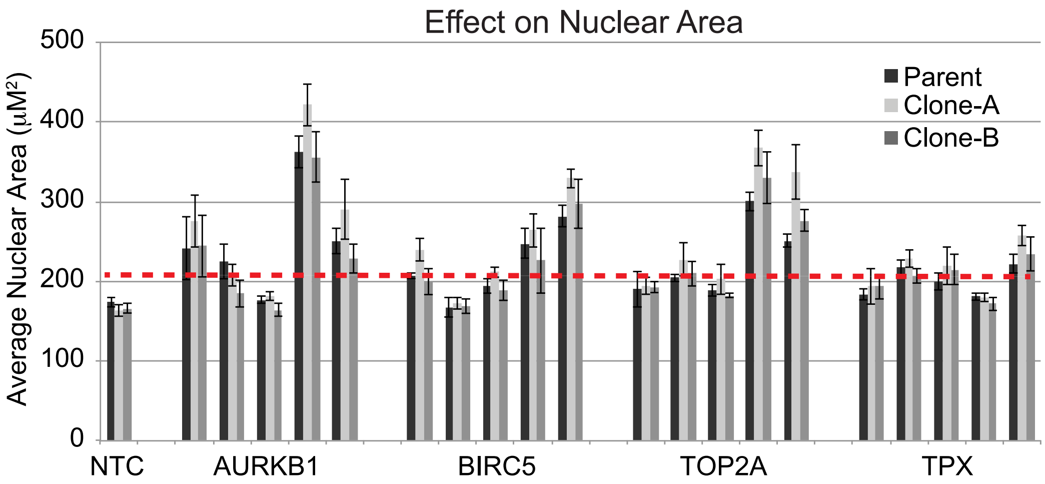

Supplement: S3 Fig — Bars represent the average and standard deviation of four replicates. The dashed line indicates five standard deviations above non-targeting control. (PDF) [file pone.0168968.s003.pdf]
