# Supplementary material for: Validation of Synthetic CRISPR Reagents as a Tool for Arrayed Functional Genomic Screening
Source: PLoS One. 2016 Dec 28;11(12):e0168968. doi: 10.1371/journal.pone.0168968 (PMC5193459; doi:10.1371/journal.pone.0168968)

Supplemental Figure 4. Comparison of the effect of different guides (left) or siRNAs (right) targeting the same gene on measured nuclear area.

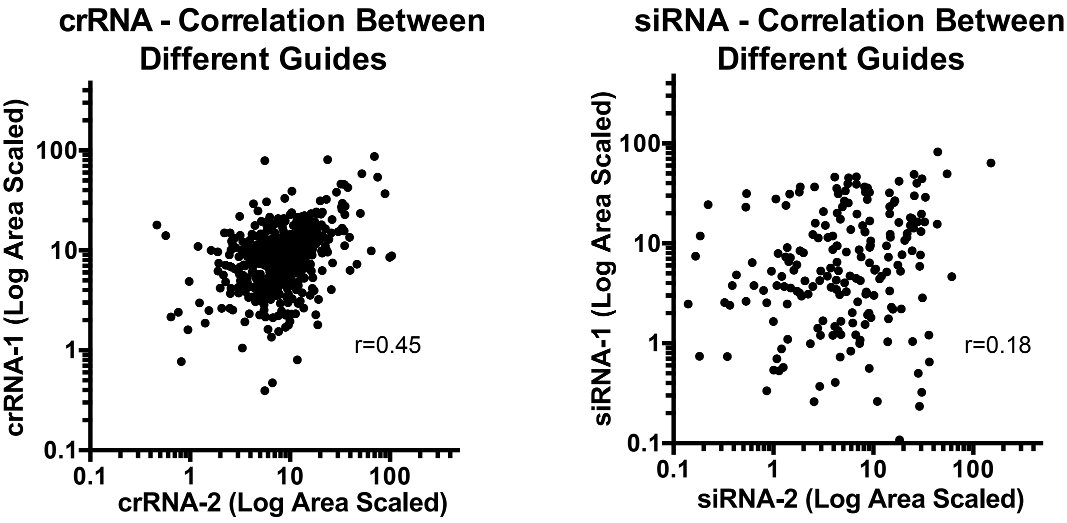

Supplement: S4 Fig — (PDF) [file pone.0168968.s004.pdf]
